# The Divergent Intracellular Lifestyle of *Francisella tularensis* in Evolutionarily Distinct Host Cells

**DOI:** 10.1371/journal.ppat.1005208

**Published:** 2015-12-03

**Authors:** Mateja Ozanic, Valentina Marecic, Yousef Abu Kwaik, Marina Santic

**Affiliations:** 1 Department of Microbiology and Parasitology, University of Rijeka, Medical faculty, Rijeka, Croatia; 2 Department of Microbiology and Immunology and Center for Predictive Medicine, College of Medicine, University of Louisville, Louisville, Kentucky, United States of America; University of North Carolina at Chapel Hill School of Medicine, UNITED STATES

## Overview

*Francisella tularensis* is a gram-negative, facultative, intracellular bacterium that survives in mammals, arthropods, and amoebae; however, macrophages are considered the key cells in pathogenesis of tularemia in mammals. Understanding intracellular trafficking of *F*. *tularensis* within various host cells is indispensable to our understanding of bacterial ecology, intracellular adaptation to various hosts’ microenvironments, and subversion of host cell defenses. Within mammalian and arthropod-derived cells, *F*. *tularensis* transiently resides within an acidic vacuole prior to escaping to the cytosol, where the bacteria replicate. In contrast, *F*. *tularensis* resides and replicates within non-acidified, membrane-bound vacuoles within the trophozoites of amoebae. The *Francisella* pathogenicity island (FPI) genes encode a type VI Secretion System (T6SS), which is indispensable for phagosomal escape of *F*. *tularensis* within mammalian and arthropod cells and for intravacuolar growth within amoeba. In this review, we discuss the divergent *F*. *tularensis* intracellular lifestyle in different hosts and its role in pathogenic evolution and intracellular proliferation within diverse hosts.

## Introduction

Nonpathogenic bacteria are taken up by host cells into vacuoles or phagosomes that are processed through the endocytic pathway, through which the vacuoles mature and fuse to the lysosomes, in which the bacteria are degraded. To avoid this fate within phagocytic cells, intracellular pathogens have evolved different strategies to survive and evade phagosome–lysosome fusion [1]. Understanding the mechanisms by which pathogens manipulate vesicle trafficking in different hosts is extremely important for understanding the ability of various pathogens to cause disease and is essential for designing novel and effective strategies for prevention and therapeutic intervention.

*Francisella tularensis* is a gram-negative, highly infectious, facultative intracellular bacterium that causes the zoonotic disease tularemia. The genus *Francisella* contains five species: *F*. *tularensis*, *F*. *philomiragia*, *F*. *hispaniensis*, *F*. *noatunensis*, and *F*. *novicida* [2,3]. Two subspecies of *F*. *tularensis*, *tularensis* (Type A) and *holarctica* (Type B), cause most cases of the illness in humans. *F*. *novicida* causes disease only in immunocompromised persons, but is highly virulent in mice [3]. However, it is important to note that in comparison to *F*. *tularensis* subsp. *tularensis* and *F*. *tularensis* subsp. *holarctica*, *F*. *novicida* elicits a different immune response in the host [2].

Humans acquire infection by several routes, including direct contact with infected animals, ingestion of water or food contaminated by infected animals, exposure to infected arthropod vectors, or by inhalation of infective aerosols, resulting in pneumonic, oropharyngeal, glandular, ulceroglandular, or oculoglandular tularemia [4]. Considering the ease of dissemination and high infectivity, *F*. *tularensis* subsp. *tularensis* and *F*. *tularensis* subsp. *holarctica* have been classified by the Centers for Disease Control and Prevention (CDC) as Tier 1 select agents.

The existence of *Francisella* in the environment is divided into two cycles: the terrestrial cycle and aquatic cycle [5]. Small rodents, hares, and arthropods play a major role in the terrestrial cycle, while rodents associated with water are important in the water cycle [4,5]. Organisms such as ticks, flies, and mosquitoes are considered vectors of tularemia transmission to mammals [6]. Although it causes disease in various animal species, no animal has been identified as a main reservoir of this pathogen. *F*. *tularensis* subsp. *holarctica* and *F*. *novicida* have a strong association with freshwater environments, free-living amoeba, and biofilms [7,8]. Since mosquito larvae can feed on aquatic protozoa, they may be infected with *F*. *tularensis* during development in their natural aquatic environment [7]. The effect of wars, natural disasters, climate change, and global warming will probably have an impact on increased incidences of tularemia [9].

## The Role of T6SS in Intracellular Replication

Understanding the virulence factors of *Francisella* is indispensable for elucidating various aspects of tularemia pathogenesis. Many studies have been focused on a genomic region called the *Francisella* pathogenicity island (FPI). The FPI is duplicated in *F*. *tularensis* subsp. *tularensis* and *F*. *tularensis* subsp. *holarctica* strains, but is a single copy in *F*. *novicida*. It has been shown that many of the FPI genes are essential for phagosome biogenesis and escape of the bacterium into the cytosol, which is the crucial event in the intracellular life cycle of *Francisella* [10].

The FPI genes encode a type VI Secretion System (T6SS), since the FPI-encoded proteins IglA, IglB, VgrG, PdpB, and DotU show similarity to structural components of T6SS of other bacteria [10,11]. The existence of the FPI-encoded secretion system is supported by finding that IglI and VgrG proteins are secreted by *F*. *novicida* to the cytosol of macrophages [10]. A lipoprotein that anchors parts of the secretion apertures to the outer membrane is commonly encoded by a T6SS gene cluster [12]. It has been recently shown that the FPI-encoded protein IglE is a lipoprotein that localizes to the outer membrane and interacts with other FPI proteins, resulting in a channel formation [13]. The T6SS assembles a phage tail-like injectisome for translocation of effector proteins across the two bacterial membranes and host cell membrane, and into the cytosol of the host cell [14]. This secretion system is involved in *F*. *tularensis* phagosomal escape and intracellular growth. However, the *Francisella* FPI system seems to be different from all other T6SS described so far, since no homolog of ClpV has been identified and the *Francisella* VgrG is significantly smaller than VgrG homologs from other bacteria [15,16].

The most investigated FPI protein, IglC, and the global regulator MglA (encoded outside the FPI) are essential for the ability of *Francisella* to escape from the *Francisella*-containing phagosome (FCP) into the cytoplasm and for pathogenesis of tularemia in vivo [17]. The FPI genes iglA, iglB, iglD, pdpA, vgrG, and iglI are also required for intracellular growth and virulence of *F*. *tularensis* [10,18]. Although the mechanism of escape of *Francisella* from the phagosome is not known yet, many of the proteins encoded by the FPI are indispensable for this process.

## Intracellular Life of *F*. *tularensis* in Macrophages

*F*. *tularensis* survives and replicates within various cells, but macrophages are considered the important cells in developing tularemia [19]. *Francisella* enters into macrophages by looping phagocytosis and binding to surface receptors, depending on the opsonization state [20]. Upon entry into macrophages, *F*. *tularensis* recruits ''lipid rafts'' (cholesterol-rich lipid domains) with caveolin-1 on the host cell membrane [21]. Cholesterol and caveolin-1 are incorporated into the FCP membrane during the initial phase of biogenesis of the FCP [22].

**Fig 1:**
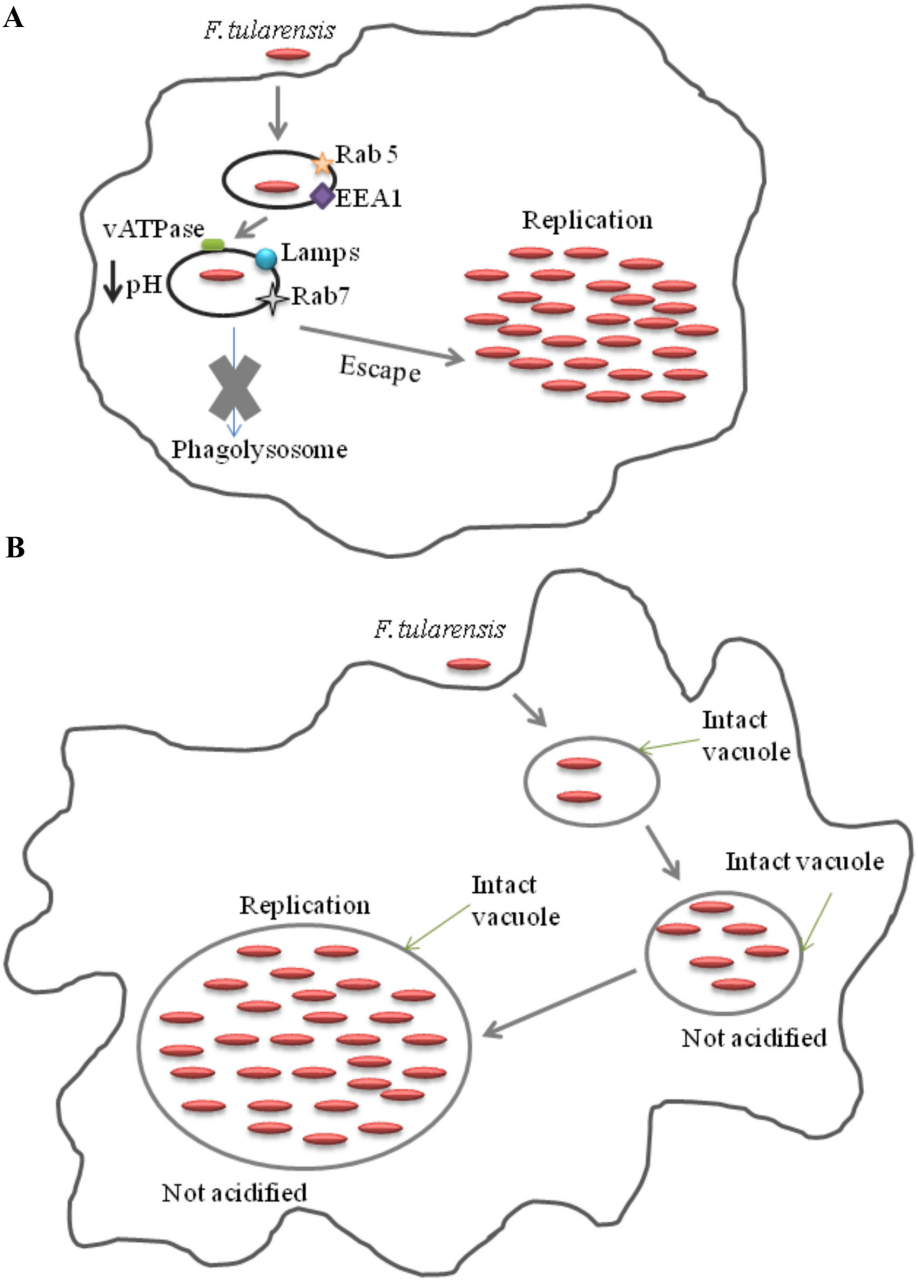
Trafficking of *Francisella* within macrophages (A) and amoebae cells (B). (A) Upon phagocytosis by macrophages, bacteria reside in the FCP that interact with early (EEA1 and Rab 5) and late (Lamps and Rab 7) endocytic compartments. The FCP is rapidly acidified by the vATPase proton pump, resulting in bacterial escape into the cytosol, where it replicates. (B) After phagocytosis by amoebae, bacteria are localized in intact vacuoles. Bacteria reside in the vacuoles and replicate.

**Fig 2:**
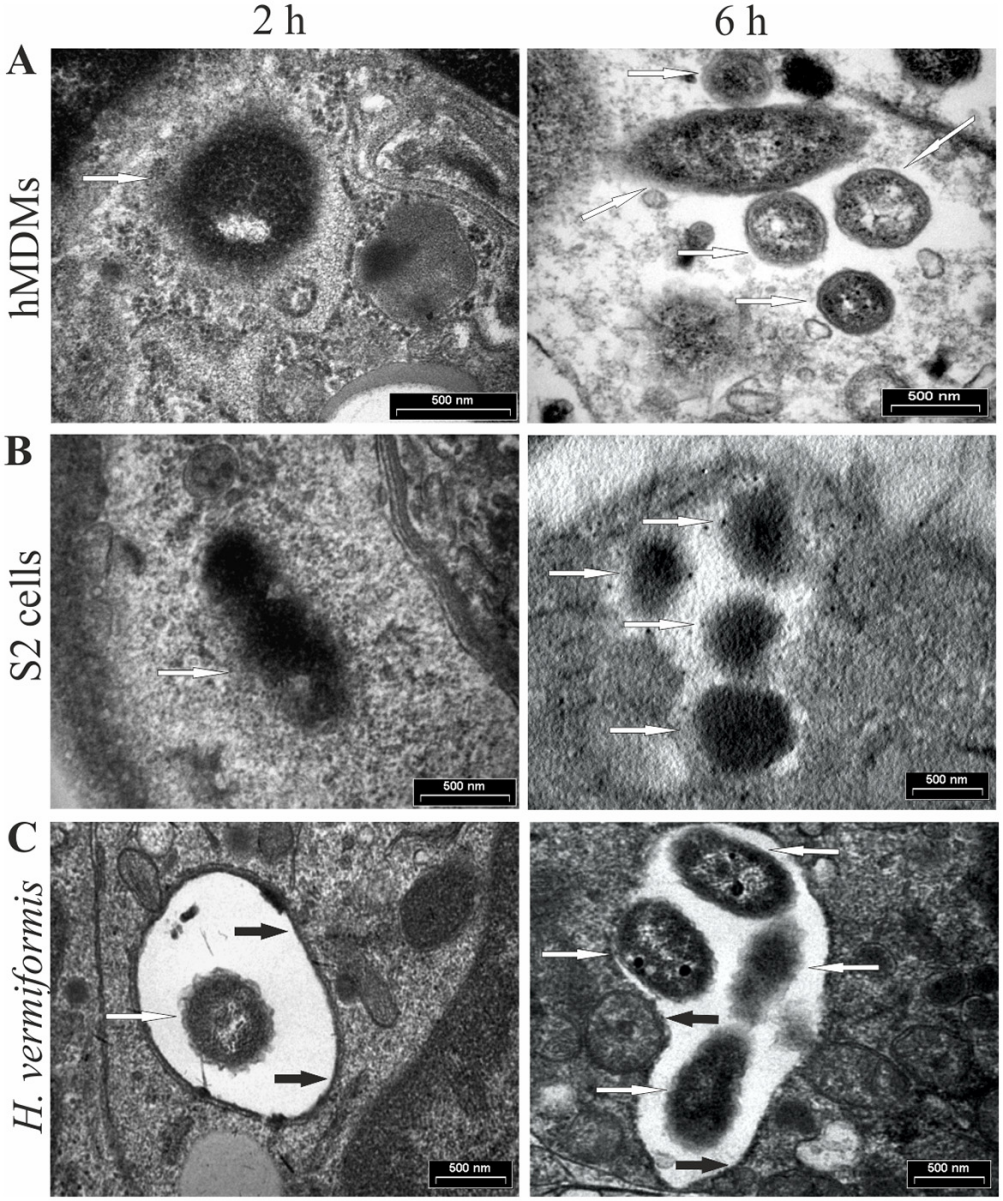
*F*. *tularensis* escapes from the phagosome and replicates in the cytosol of human macrophages (A) and *Drosophila melanogaster*-derived S2 cells (B), but resides and replicates in vacuoles within amoeba (C). Representative electron micrographs of hMDMs, *D*. *melanogaster*, and *Hartmannella vermiformis* infected with *F*. *tularensis* at 2 and 6 h after infection. The white arrows indicate bacteria, while black arrows indicate intact vacuolar membrane.

Following uptake of *Francisella* by macrophages, the bacteria reside within the FCP, which matures into an early endosome characterized by Rab-5 and early endosome antigen 1 (EEA1) [23]. The FCP matures into a late endosomal stage characterized by the late endosomal markers Rab-7, mannose-6-phosphate, and lysosomal associated proteins (LAMPs), but the FCP does not mature into a phagolysosome [17,24,25]. The FCP is acidified through acquisition of the vATPase proton pump [23,25]. This is followed by escape of the bacteria to the cytosol within 30 to 60 minutes of entry [17,25]. The short time spent in the FCP is a dynamic step in the infection during which *Francisella* actively evades host antimicrobial defenses, including reactive oxygen species and antimicrobial peptides [26]. Interestingly, inhibition of the acidification by a specific inhibitor of the vATPase proton pump, bafilomycin A, does not block, but delays, bacterial escape to the cytosol (Fig 1A) [25]. It appears that acidification of the FCP enables rapid phagosomal membrane disruption and bacterial egress in the macrophage cytosol, where they replicate (Fig 2A) [23]. It seems that a decrease in pH stimulates *Francisella* to express or secrete some unique, unknown factor to disrupt the phagosomal membrane, but the mechanisms involved are still to be determined. Late stages of intracellular proliferation are accompanied with or followed by death of infected macrophages, mediated by a versatile apoptotic pathway able to switch from pyroptosis to a mitochondrial-intrinsic apoptotic pathway [27]. It is followed by egress of intracellular bacteria [28].

## Intracellular Life of *F*. *tularensis* in Arthropod-Derived Cells

Vector-borne transmission of tularemia to mammalian hosts has an important role in pathogenesis of the disease [29]. However, little is known about the interaction of *F*. *tularensis* with the arthropod vectors at the molecular level. *Drosophila melanogaster* and *D*. *melanogaster*-derived S2 cells have been used for studying *F*. *tularensis* infection [30]. It has been shown that *F*. *tularensis* infects and kills adult *Drosophila* flies in a dose-dependent manner [31]. Similar to human macrophages, within arthropod cells, *F*. *tularensis* transiently resides within an acidified phagosome that matures to a late endosomal stage, followed by rapid bacterial escape into the cytosol, where the bacteria proliferate (Fig 2B) [32]. The intracellular lifestyle of *F*. *tularensis* within human macrophages and arthropod-derived cells is very similar in terms of the virulence factors involved. Studies using a mosquito cell line, SualB, derived from *Anopheles gambie*, have shown that the FPI-encoded IglA, IglB, IglC, IglD, PdpA, and PdpB are necessary for efficient intracellular replication of *F*. *novicida* within this insect’s cells [33]. However, the FPI proteins PdpC and PdpD are not required for replication within the SualB cell line [33]. In addition, out of 394 mutants of *F*. *novicida* identified to be defective for proliferation within S2 cells, only 135 (including the FPI genes) are also defective for replication within human macrophages [30]. *F*. *novicida* virulence factors have also been studied in adult flies compared to mice [34]. Among 249 *F*. *tularensis* genes important for virulence in the mice model, only 49 genes were important in adult *D*. *melanogaster*, including 14 of the FPI genes [34]. The FPI genes that are not required for virulence in the adult fly are *pdpC*, *pdpE*, *pdpD*, and *anmK* [34], which is consistent with findings in the mosquito cell line [33]. Therefore, although *F*. *tularensis* uses similar molecular mechanisms of pathogenesis within arthropod and mammalian cells, some distinct virulence factors are differentially utilized for bacterial proliferation in the two evolutionarily distinct hosts [30].

## Intravacuolar Proliferation of *F*. *tularensis* in Amoeba

Recent studies have shown that *Francisella* enters and multiplies within *Acanthamoeba castellanii* [35], *Hartmannella vermiformis* [36], and *Dictyostelium discoideum* cells (our unpublished results). In addition, *F*. *tularensis* subsp. *holarctica* is found within cysts of *A*. *castellanii*, suggesting a potential for long-term bacterial survival in nature [37]. Studies have also shown intravacuolar replication of *F*. *novicida* within *H*. *vermiformis* (Figs 1B and 2C), which is a major difference from the cytosolic proliferation of this bacterium within mammalian and arthropod-derived cells. Interestingly, the FPI-encoded protein IglC and the MglA global regulator, which are essential for phagosomal escape and intramacrophage growth of *F*. *tularensis*, are also important for intravacuolar replication of *F*. *tularensis* within amoeba cells [36,38].

In addition, imaging studies using the lysosomotropic agent LysoTracker Red DND-99, which concentrates in acidified vesicles and compartments, have shown that FCP did not acquire this dye at any time point during the infection of amoebae with *F*. *novicida* [36]. In addition, *F*. *novicida* blocks lysosomal fusion to the FCP within *A*. *castellanii* [37]. Therefore, *F*. *tularensis* escapes from acidified vacuoles in human and arthropod-derived cells, but replicates within non-acidified vacuoles in *H*. *vermiformis* [36]. Although the FPI protein IglC plays an important role in intravacuolar proliferation of *F*. *tularensis* within protozoa, it is not sufficient to trigger bacterial escape into the cytosol. It is unclear how IglC contributes to phagosomal escape in human and arthropod-derived cells but is also required for intravacuolar growth of *F*. *tularensis* within amoeba. It is also unclear why *F*. *tularensis* within amoeba has a different intracellular cycle compared to arthropod and mammalian cells.

## Conclusions and Future Directions

The ability to invade and replicate in a variety of host cells appears to be a major feature of the ecology and epidemiology of *F*. *tularensis*. Within both mammalian and arthropod-derived cells, the FCP transiently matures to an acidified late endosome, followed by rapid bacterial escape into the host cell cytosol where the replication occurs. However, within amoeba cells, the bacterium resides and replicates within non-acidified, membrane-bound vacuoles. It is possible that the virulence of *Francisella* for mammalian hosts may be higher after intra-amoebal growth. Many virulence factors of *F*. *tularensis* have been discovered and investigated, including the FPI proteins, which play a crucial role in patho-adaptation of the bacteria to different hosts. At the moment, some studies are focused on the FPI genes that encode the T6SS in *Francisella*. Future studies should elucidate the role of T6SS translocated proteins in disruption of the phagosome and intracellular replication of *F*. *tularensis*. The relevance of bacterial escape into the cytosol should be investigated from various perspectives and cells models. It is evident that the short transition of the bacterium in vacuoles within mammalian and arthropod cells plays an important role in pathogenesis of tularemia. The differences between the amoebal vacuoles and the mammalian and arthropod vacuoles where the bacterium permanently or shortly resides, respectively, have yet to be discovered. It is intriguing how IglC is required for phagosomal escape in mammalian and arthropod-derived cells but is indispensable for intravacuolar growth within amoeba. It is possible that the long-term evolution of *F*. *tularensis* within amoeba has facilitated its intravacuolar adaptation in the aquatic environment for long-term survival and for transmission to arthropod and mammalian hosts.
